# Assessment of the efficiency of different chemical treatments and ultrasonic cleaning for defatting of cancellous bone samples

**DOI:** 10.1007/s10561-021-09969-x

**Published:** 2021-10-29

**Authors:** Fangxing Wang, Florian Metzner, Georg Osterhoff, Stefan Schleifenbaum

**Affiliations:** 1 ZESBO - Center for Research on Musculoskeletal Systems, Department of Orthopaedic Surgery, Traumatology and Plastic Surgery, Leipzig University, Semmelweisstraße 14, 04103 Leipzig, Germany; 2 Department of Orthopedic Surgery, Traumatology and Plastic Surgery, Leipzig University, Liebigstraße. 20 Haus 4, 04103 Leipzig, Germany

**Keywords:** Defatting, Cancellous bone, Trabecular bone, Bone marrow, Ultrasonic cleaning, Chemical treatments

## Abstract

Our study aimed to asses the defatting efficiency of different methods, which are commonly used and easily available in the laboratory in order to find a method that is effective, convenient, safe, and economical. Cylindrical cancellous bone specimens were obtained from fresh-frozen human cadaver femoral condyles, cut into multiple small specimens (Ø8 × 2 mm), and assigned to two groups that were treated with either chemical solvent soaking (Solvent group) or ultrasonic cleaning (Ultrasound group). Each group was divided into several subgroups based on different treatments. Digital photographs were taken of each specimen. The difference of material density (Δρ_b_), apparent density (Δρ_app_), and porosity (ΔP) before and after treatment were used as evaluation indicators. For the solvent group, in Δρ_b_, only the combination of 99% ethanol and detergent solution showed a significant difference before and after treatment (*P* = 0.00). There was no significant difference in ΔP among acetone, the mixture of 99% ethanol and acetone, and the combination of 99% ethanol and detergent solution (*P* = 0.93). For the ultrasound group, the median of all subgroups in Δρ_app_ and ΔP were all lower than the solvent group. The combination of 99% ethanol and detergent solution (v/v = 1:20), as well as the mixture of 99% ethanol and acetone (v/v = 1:1), seem to be the optimal defatting methods for 2 mm thick cancellous bone slices due to their effectiveness, availability, low-cost and safety. Chemical soaking for 24 h is more effective than ultrasonic cleaning with 99% ethanol or acetone for 20 or 40 min.

## Introduction

Bone marrow fat (BMF) accounts for approximately 70% of adult bone marrow volume (Wang et al. 2018) and even more in elderly patients with osteoporosis (Yeung et al. 2005). In order to accurately obtain density values of cancellous bone samples, defatting is a critical procedure to prevent the effects from bone marrow or lipids (Sharp et al. 1990). The concept of defatting is relatively simple but selecting an appropriate and effective method for cancellous bone specimens is an issue worth considering. Cancellous bone is relatively fragile, especially for elderly patients with osteoporosis. Inappropriate defatting methods may affect the mechanical properties of the specimens and even cause damage.

Although the high-pressure air/water jet is a physical defatting method commonly used in the laboratory (Hua et al. 2020; Zioupos et al. 2008), it has certain limitations when used for small specimens. High-pressure air/water from the jet easily can cause bone slices to scatter. Besides, it probably causes contamination of the laboratory environment in the absence of a ventilation system around. Lipase is also regarded as a promising substance for defatting of cancellous bone in the bone graft, in a shorter time and without toxic effects (Zhang et al. 2014). The effectiveness and reliability have been confirmed in the porcine bone experiment (Zhang et al. 2014). However, the activity of lipase strongly depends on pH and temperature, which leads to difficult control in practice (Gardin et al. 2015). Another study proposed a novel method that uses supercritical fluid extraction to defat based on the principle of extraction (Fages et al. 1994). The use of supercritical CO_2_ in defatting has been proved efficient and safe, it does not involve toxic chemicals and pollution to the environment. Thus, this treatment can be used in bone grafts (Fages et al. 1994). However, expensive equipment and complex operations limit the widespread promotion.

Ultrasonic cleaning has been used in industry for decades, especially for cleaning of small and complex parts, which can accelerate the surface treatment process (Kieser et al. 2011). It’s also a common way for defatting of cancellous bone specimens (Shao et al. 2010; Sharp et al. 1990). A study on the efficiency of different chemical reagents (absolute alcohol and trichloroethylene) combined with an ultrasound bath. Especially small cancellous bone samples are commonly defatted using chemical solvent soaking and ultrasonic cleaning (Sharp et al. 1990).

The purpose of this study was to evaluate the defatting efficiency of different chemical solvents soaking and ultrasonic cleaning methods for defatting of cancellous bone specimens. The goal was to identify an effective, convenient, easy-accessible, and safe defatting method.

## Materials and methods

**Fig. 1:**
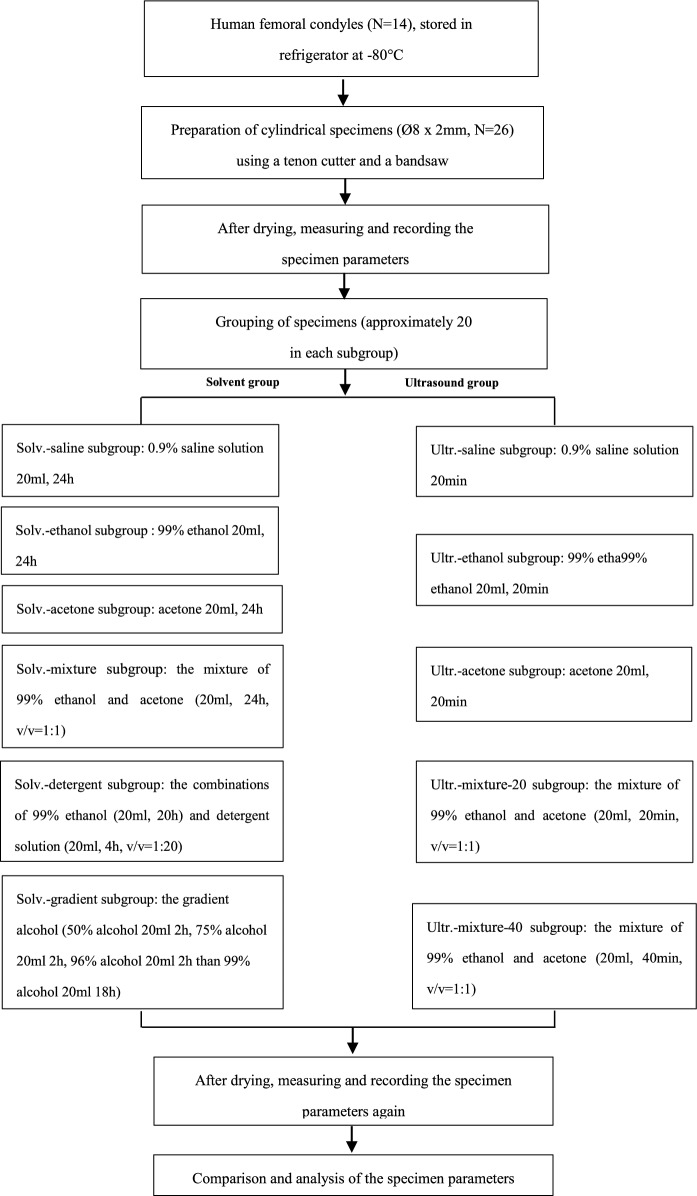
The flow chart for the detailed steps of our experiment

The experiment was separated into two stages: (i) before formal testing, a pre-test was performed to determine the drying time of different reagents at room temperature (21 °C), which is essential for shortening time and improving experimental efficiency. (ii) The bone slices were divided into different groups to compare the efficiency and advantages of varying defatting methods. The detailed steps can be seen in the flowchart (Fig. 1).

### Donors

Fourteen femoral condyles obtained from nine human body donors with a mean age of 85.4 years (range: 74–97 years, 5 males and 4 females) were collected and stored fresh-frozen at −80 °C until further testing. All body donors gave their informed and written consent to the donation of their bodies for teaching and research purposes while alive. Being part of the body donor program is regulated by the Saxonian Death and Funeral Act of 1994 (third section, paragraph 18 item 8), institutional approval for the use of the post-mortem tissues of human body donors was obtained from the Institute of Anatomy, University of Leipzig. The authors declare that all experiments were conducted according to the principles of the Declaration of Helsinki.

### Specimens preparation

**Fig. 2:**
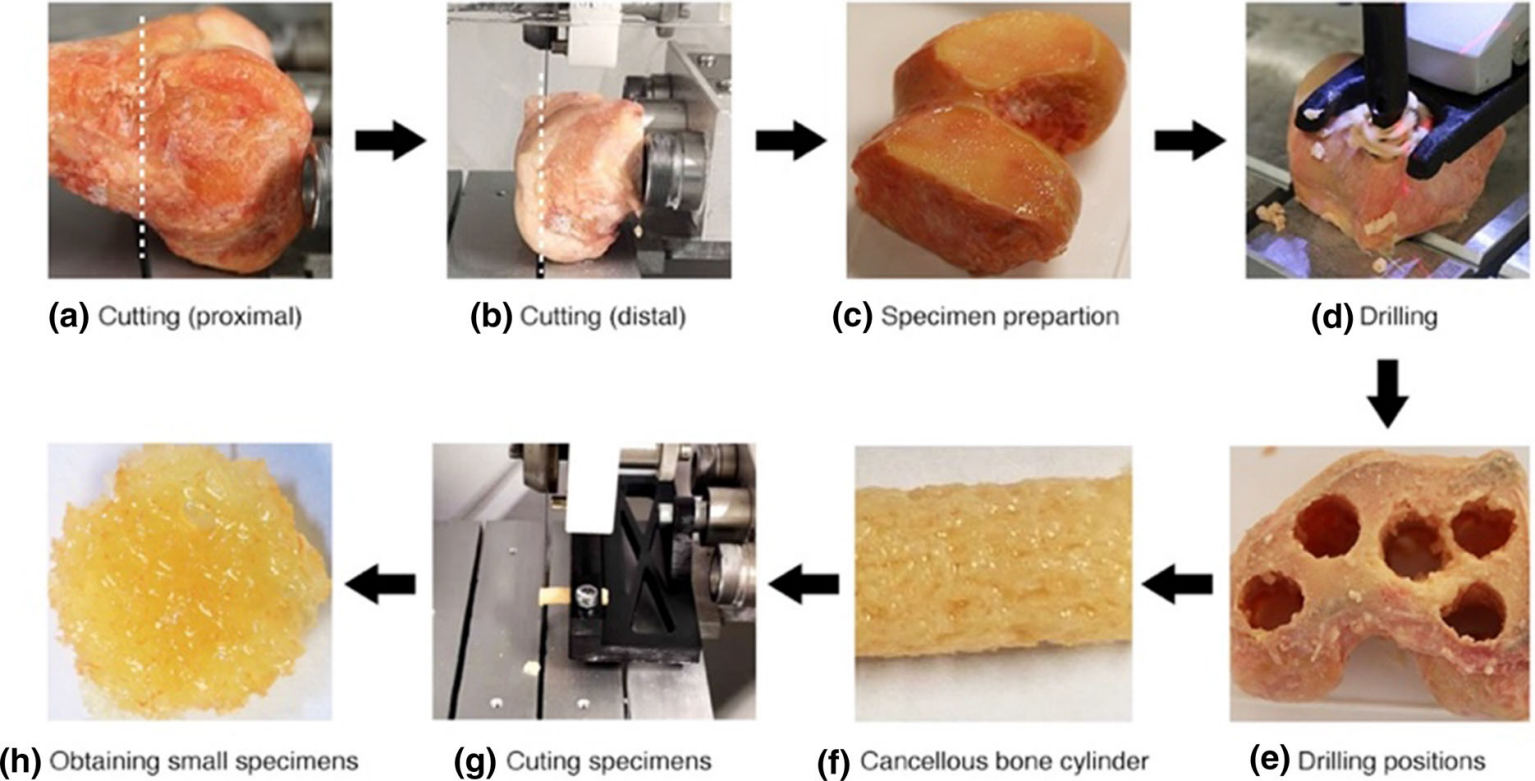
The steps of specimen preparation obtained from human femoral condyle

Cylindrical cancellous bone specimens were collected from femoral condyles using a tenon cutter (FAMAG Series 1616, FAMAG-Werkzeugfabrik GmbH & Co. KG, Remscheid, Germany) with 8 mm internal diameter using a stationary drilling machine (model PBD 40; Robert Bosch GmbH Power Tools, Leinfelden-Echterdingen, Germany). A total of twenty-six cylindrical drill cores were wrapped in plastic foil and stored in the refrigerator at −20 °C. These cylindrical drill cores were assigned to each group randomly and equally. Then, these cylindrical specimens were cut to 2 mm thickness (Ø8 × 2 mm) using a diamond band saw (EXAKT 310, EXAKT Advanced Technologies GmbH, Norderstedt) (Fig. 2). All frozen specimens were thawed in 0.9% saline solution at room temperature (21°C). The evaluation indicators of each specimen were measured in a completely dry state.

### Pre-tests

Prior to formal testing, the drying time of the specimens immersed in different chemical solutions was tested firstly. The results showed that the drying time was as follows: 3 h was sufficient for 0.9% saline solution or detergent solution, whereas 30 min was enough for 99% ethanol or acetone. About the drying process, specimens were put on blotting paper and changed three times in order to remove inter-trabecular water until the weight measured by the precision scale was consistent (ML303T/00, Mettler-Toledo GmbH, Switzerland). The procedure of drying specimens is applied in order to avoid moisture interference between different measurement points, which is a crucial step to minimize deviations.

**Fig. 3:**
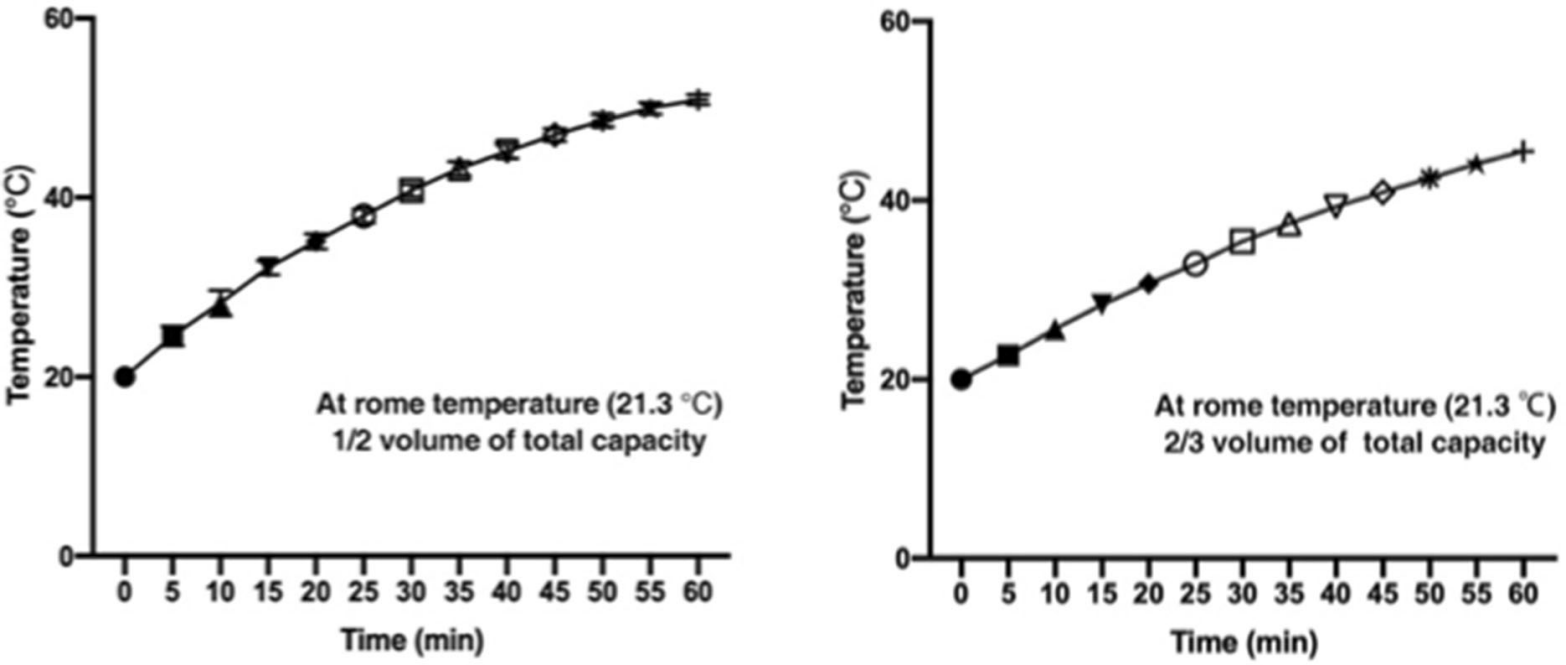
The increase of water temperature within the tank without a cover in the process of using the ultrasonic machine

Additionally, the increase in water temperature during the ultrasonic activity was tested and recorded in order to avoid potential damage to the specimens caused by excessive temperatures. The results demonstrated that the water temperature within the tank without a cover would increase even if the heater was not in operation during ultrasonic cleaning. For one hour, the water temperature rises about 30 °C when the amount of water is 1/2 of the total volume, around 25 °C for 2/3 of the total volume (Fig. 3). Therefore, during formal testing, the starting water temperature within the tank was set between 21 and 23 °C to avoid excessive water temperature at the end of one cycle (20 min).

### Group assignment

The prepared specimens were randomly and equally assigned to two groups and the following different treatments: (i) solvent group, following a process that removes the lipids based on the several chemical solvents for 24 h; (ii) ultrasound group, following a procedure on the basis of different chemical solvents combined with ultrasonic cleaning (20 min and 40 min) (Table 1).

**Table 1:** Summary of treatment methods for each subgroup

	Sub-groups	Numbers	Treatment methods
Solvent group	Solv.-saline	20	0.9% saline solution 20 ml, 24 h
	Solv.-ethanol	18	99% ethanol 20 ml, 24 h
	Solv.-acetone	21	Acetone 20 ml, 24 h
	Solv.-mixture	17	The mixture of 99% ethanol and acetone (20 ml, 24 h, v/v = 1:1)
	Solv.-detergent	19	The combinations of 99% ethanol (20 ml, 20 h) and detergent solution (20 ml, 4 h, v/v = 1:20)
	Solv.-gradient	23	The gradient alcohol (50% alcohol 20 ml 2 h, 75% alcohol 20 ml 2 h, 96% alcohol 20 ml 2 h than 99% alcohol 20 ml 18 h)
Ultrasound group	Ultr.-saline	20	0.9% saline solution 20 min
	Ultr.-ethanol	21	99% ethanol 20 ml, 20 min
	Ultr.-acetone	21	Acetone 20 ml, 20 min
	Ultr.-mixture-20	21	The mixture of 99% ethanol and acetone (20 ml, 20 min, v/v = 1:1)
	Ultr.-mixture-40	19	The mixture of 99% ethanol and acetone (20 ml, 40 min, v/v = 1:1)

99% ethanol, acetone, detergent solution (v/v = 1:20), and gradient alcohol were used in the solvent group as these reagents are known to be effective, easily available, and of low cost. The total processing time of each group was set to 24 h referring to previously published literature (Thorén et al. 1993; Zhang et al. 2014).

For the ultrasound group, the processing time was set to 20 min and 40 min in combination with different solvents (Table 1).

### Defatting process

#### Chemical solvent soaking treatment

Processing with solv.-saline, ethanol, acetone, mixture subgroups: first, the prepared specimens (2 mm thick cancellous bone slices) were placed on blotting paper in the air at room temperature (21 °C) for drying (3 h), then the parameters of each specimen were measured and recorded. Next, these specimens were put in sealable glass bottles containing different kinds of reagent solutions (20 ml) at room temperature (21 °C) for 24 h, respectively. After 24 h, the specimens were dried according to the pre-tests section (this drying procedure would be used for all following subgroups), then the parameters of each specimen were tested and recorded (Fig. 1).

For solv.-detergent subgroup, the detergent solution (v/v = 1:20) was first made using a detergent (Ja, Dishwasher detergent, Beromin GmbH, Germany) and deionized water. The difference from the process of solv.-saline, ethanol, acetone, mixture subgroups was that the specimens were put in 99% ethanol for 20 h, then removed to detergent solution for 4 h.

The treatment of solv.-gradient subgroup referred to Hua et al. (2020). Different gradients of alcohol were used (50% alcohol 20 ml 2 h, 75% alcohol 20 ml 2 h, 96% alcohol 20 ml 2 h than 99% alcohol 20 ml 18 h). In this study, the treatment time in the last step was increased to 18 h in order to be consistent with previous groups. Also, instead of 95% and 100% ethanol, we used 96% and 99% ethanol assuming it will not significantly change the effectiveness.

#### Ultrasonic cleaning treatment

First, the specimens were put in sealable glass bottles containing different chemical reagents (20 ml), then the bottles were placed in the ultrasonic machine. Timer was set to 20 min (ultr.-ethanol, ultr.-acetone, ultr.-mixture-20 subgroups) and 40 min (ultr.-mixture-40 subgroup). The starting water temperature was set at 23 °C for 0.9% saline solution and 21 °C when testing with 99% ethanol or acetone. For ultr.-mixture-40 subgroup, we changed the water once at the end of one cycle (20 min) to avoid excessive water temperature (Fig. 1).

### Data acquisition

#### Macroscopic appearance

The surface, color, and pores of the specimens were observed. Each specimen was photographed using a digital camera (Canon EOS 70D) before and after treatment. The clearer the pores and the whiter the color of the specimen, the more effective the treatment proved.

#### Measurements

The diameter and thickness of each specimen were measured with calipers (accuracy 0.02 mm) to calculate a total volume (*V*_*0*_). The parameters of each specimen were recorded and calculated, including mass in the air (*m*_*air*_), mass in submission (*m*_*sub*_), material density(ρ_*b*_), material volume (*V*_*mate*_). Masses were measured by use of a precision scale (ML303T/00, Mettler-Toledo GmbH, Switzerland) either in the air (*m*_*air*_) and in submersion (*m*_*sub*_) using a liquid of known density (ρ, 99% ethanol, relative density ~ 0.79 g/cm^3^) (Zou et al. 1997). Based on these test parameters, we calculated:

1 $${\text{Density}}:\;\rho = m/v$$

2 $${\text{Total }}\;{\text{volume}}:\;V_{0} = \pi \left( {d/2} \right)^{2} h$$

3 $${\text{Apparent }}\;{\text{density}}:\;\rho_{app} = m_{air} /v_{0}$$

4 $${\text{Material }}\;{\text{density}}:\;\rho_{b} = \rho m_{air} /(m_{air} - m_{sub} )$$

5 $$Porosity \, \left( \% \right):\;P = 100(1 - \rho_{app} /\rho_{b} )$$

### Statistical analysis

Statistical analysis was performed using SPSS 23.0 (Statistical Package for Social Science, IBM, USA). Continuous variables (ρ_b_, ρ_app_, P) were expressed as mean ± standard deviation (X̄±SD). When normal distribution could not be confirmed, the *Mann–Whitney-U test* was used to compare the difference in two independent groups, and the *Kruskall-Wallis H test* was performed to compare multiple groups. For continuous variables that conform to a normal distribution were assessed by *paired t-test* (same group) and *independent t-test* (two groups). A *one-way ANOVA test* was used for comparison between multiple groups. The significant coefficient was set at *P* < 0.05.

## Results

### Macroscopic appearance

**Fig. 4:**
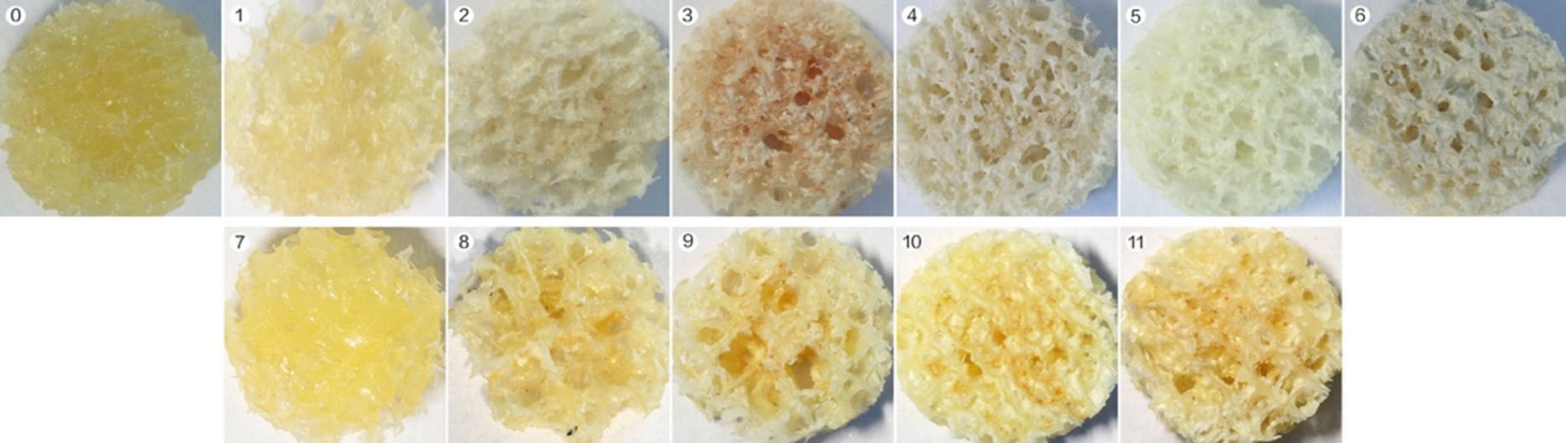
The appearance of fresh specimens and defatted specimens. The first line from left to right (Solvent group), fresh dried specimen (0), Solv.-saline subgroup (1), Solv.-ethanol subgroup (2), Solv.-acetone subgroup (3), Solv.-mixture subgroup (4), Solv.-detergent subgroup (5), Solv.-gradient subgroup (6). The second line from left to right (Ultrasound group), Ultr.-saline subgroup (7), Ultr.-ethanol subgroup (8), Ultr.-acetone subgroup (9), Ultr.-mixture-20 subgroup (10), Ultr.-mixture-40 subgroup (11). The fresh dried specimen, Solv.-saline subgroup, and Ultr.-saline subgroup were yellow with a lot of fat in the pores, the specimens that defat by chemical solvent soaking for 24 h were white and the pores were relatively clean, the specimens that defat by ultrasonic cleaning (20 min or 40 min) still leave a certain amount of lipids in specimens

Before treatment, the surface of the specimen was yellow, covered with fat, and the pores were filled with bone marrow. The specimens treated with 0.9% saline solution still had a certain amount of lipids left. However, the specimens defatted by other methods appeared white and the pore walls were relatively clean without soft tissue adhesion (Fig. 4).

### Measurement of the material density (ρ_b_)

**Fig. 5:**
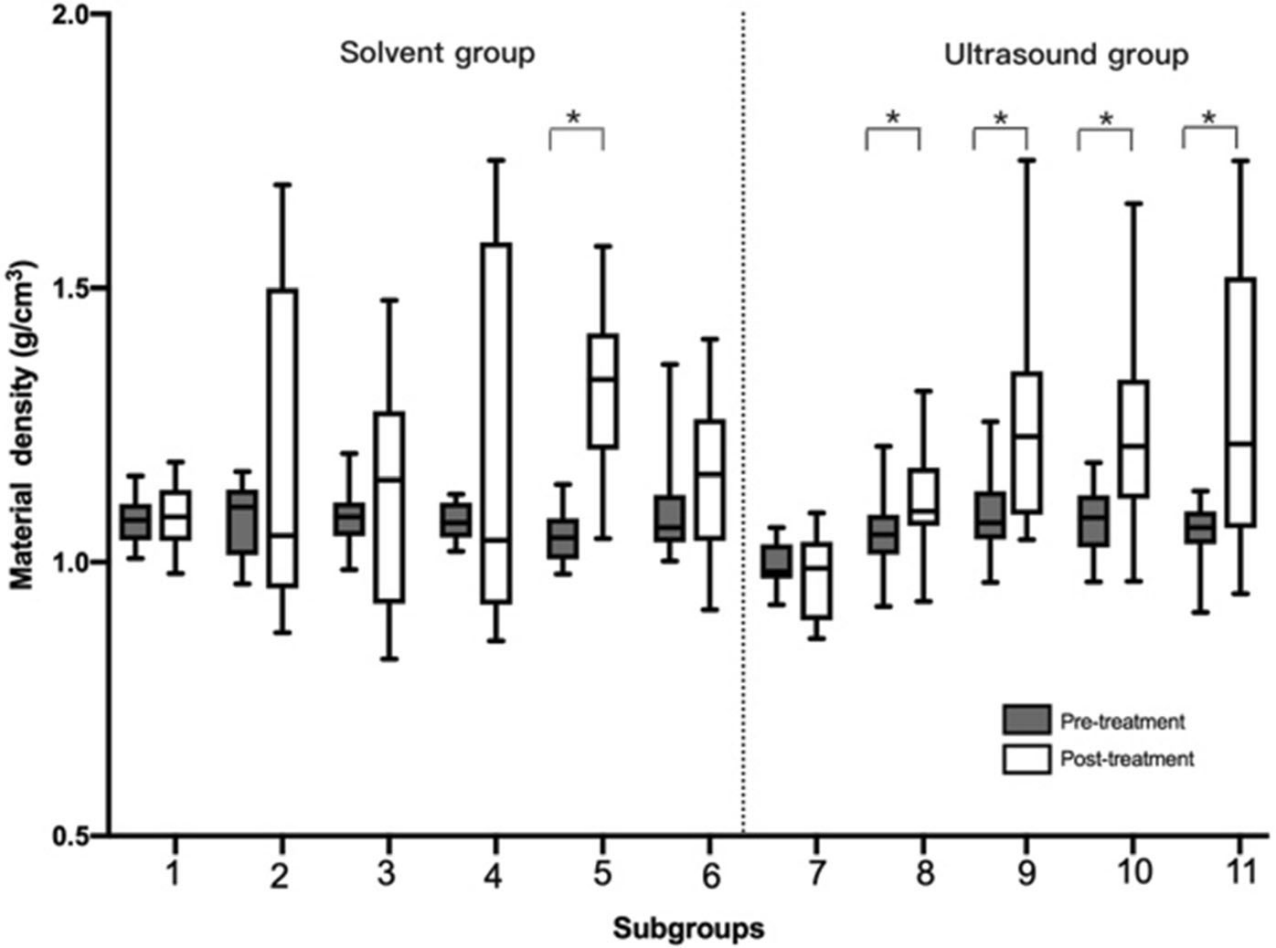
The difference in material density (ρ_b_) among subgroups pre- and post-treatment (*P < 0.05). Subgroup 1 (solv.-saline subgroup), Subgroup 2 (solv.-ethanol subgroup), Subgroup 3 (solv.-acetone subgroup), Subgroup 4 (solv.-mixture subgroup), Subgroup 5 (solv.-detergent subgroup), Subgroup 6 (solv.-gradient subgroup), Subgroup 7 (ultr.-saline subgroup), Subgroup 8 (ultr.-ethanol subgroup), Subgroup 9 (ultr.-acetone subgroup), Subgroup 10 (ultr.-mixture-20 subgroup), Subgroup 11 (ultr.-mixture-40 subgroup)

The median value of solv.-detergent subgroup was higher than that of all the rest of the subgroups in Δρ_b_ (Fig. 5). Furthermore, for the solvent group, there was a significant difference in ρ_b_ before and after treatment for solv.-detergent subgroup (*P* = 0.00), whereas there was no significant difference for the rest of the subgroups (*P* > 0.05). For the ultrasound group, there was no significant difference in ρ_b_ before and after treatment for ultr.-saline subgroup (*P* = 0.12), whereas there was a significant difference for the remaining subgroups (*P* < 0.05) (Figs. 5, 8).

#### Measurement of the apparent density (ρ_app_)

For the solvent group, there was a significant difference in Δρ_app_ between the remaining solv.-ethanol, acetone, mixture, detergent, gradient subgroups and the solv.-saline subgroup (*P* = 0.00).

**Fig. 6:**
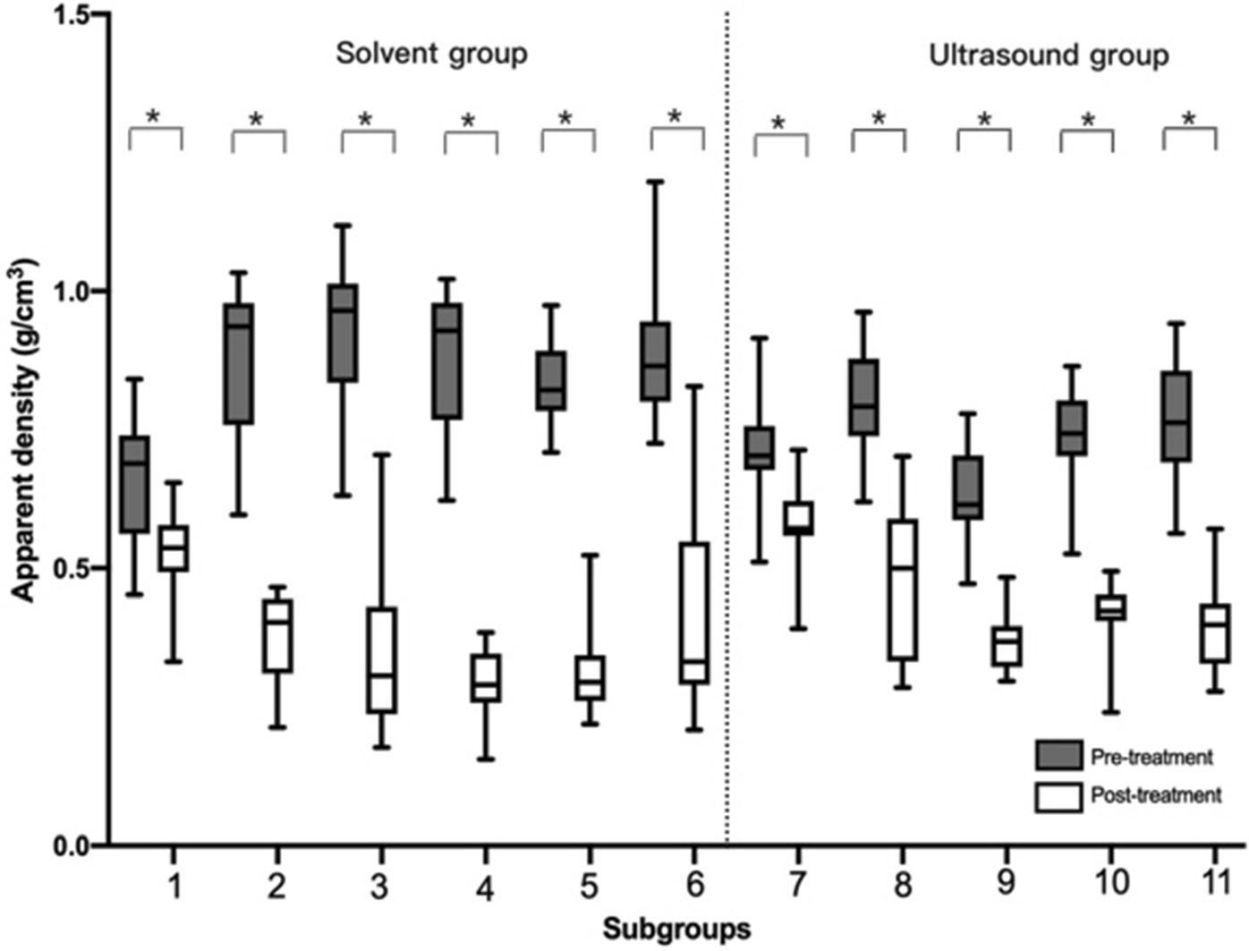
The difference in apparent density (ρ_app_) among subgroups pre- and post-treatment (*P < 0.05). Subgroup 1 (solv.-saline subgroup), Subgroup 2 (solv.-ethanol subgroup), Subgroup 3 (solv.-acetone subgroup), Subgroup 4 (solv.-mixture subgroup), Subgroup 5 (solv.-detergent subgroup), Subgroup 6 (solv.-gradient subgroup), Subgroup 7 (ultr.-saline subgroup), Subgroup 8 (ultr.-ethanol subgroup), Subgroup 9 (ultr.-acetone subgroup), Subgroup 10 (ultr.-mixture-20 subgroup), Subgroup 11 (ultr.-mixture-40 subgroup)

For the ultrasound group, the median Δρ_app_ was lower than the solvent group. There was a significant difference in Δρ_app_ among ultr.-ethanol, acetone, mixture-20, mix-40 subgroups with the ultr.-saline subgroup (*P* = 0.00). There was no significant difference in Δρ_app_ between solv.-saline subgroup and ultr.-saline subgroup (*P* = 0.71). There was a significant difference in the Δρ_app_ between ultr.-mixture-20 subgroup and ultr.-mixture-40 subgroup (*P* = 0.00), (Figs. 6, 8).

### Measurement of the porosity (P)

**Fig. 7:**
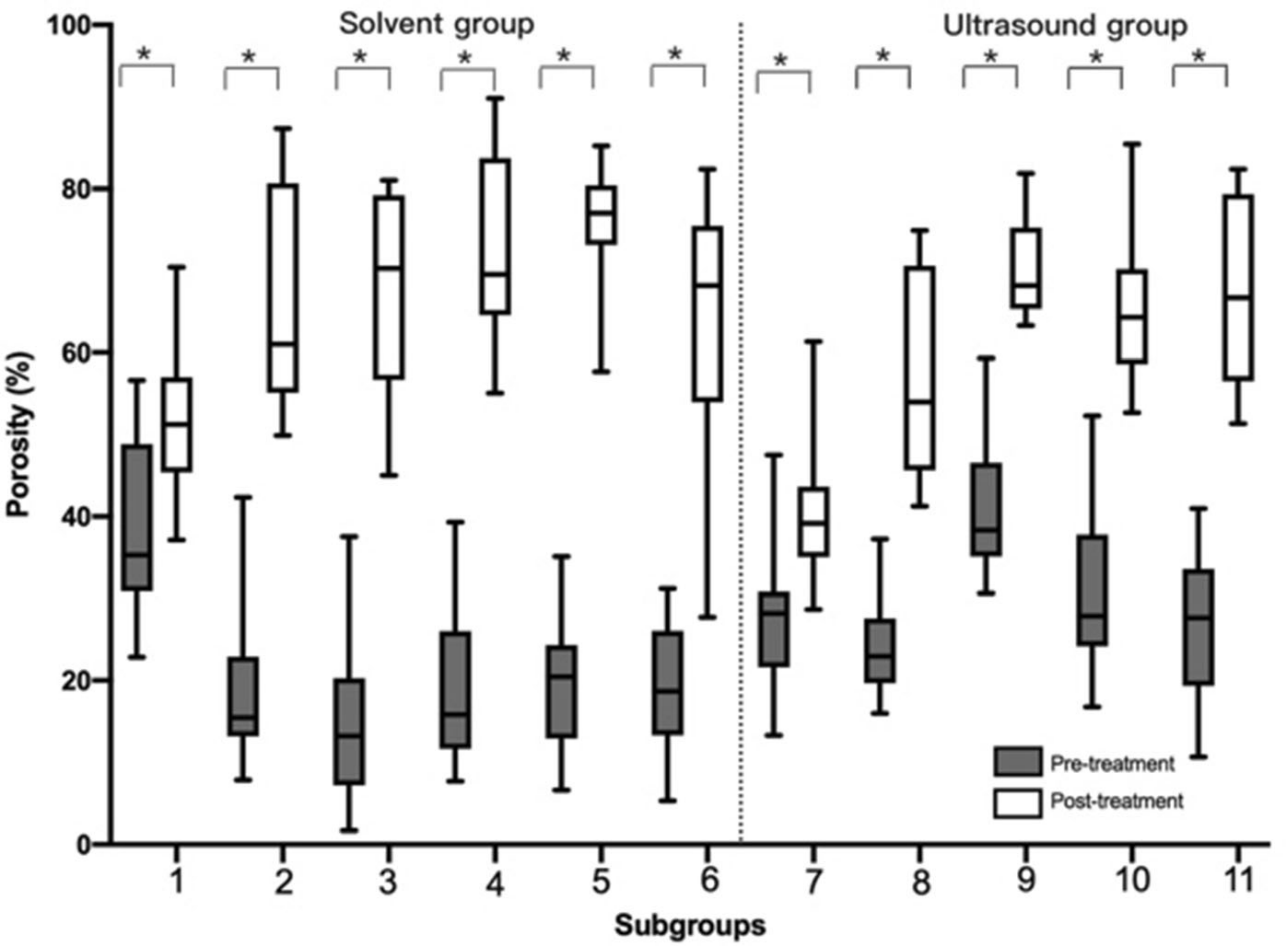
The difference in porosity (P) among subgroups pre- and post-treatment (*P < 0.05). Subgroup 1 (solv.-saline subgroup), Subgroup 2 (solv.-ethanol subgroup), Subgroup 3 (solv.-acetone subgroup), Subgroup 4 (solv.-mixture subgroup), Subgroup 5 (solv.-detergent subgroup), Subgroup 6 (solv.-gradient subgroup), Subgroup 7 (ultr.-saline subgroup), Subgroup 8 (ultr.-ethanol subgroup), Subgroup 9 (ultr.-acetone subgroup), Subgroup 10 (ultr.-mixture-20 subgroup), Subgroup 11 (ultr.-mixture-40 subgroup)

**Fig. 8:**
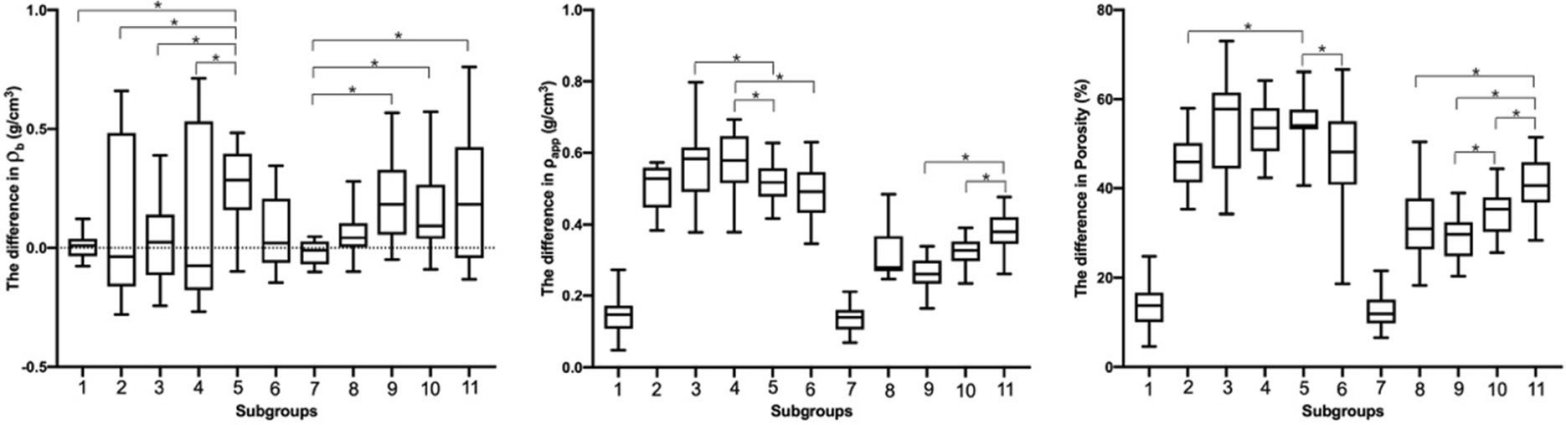
The difference of material density (ρ_b_), apparent density (ρ_app_), and porosity (P) in all subgroups (*P < 0.05). Subgroup 1 (solv.-saline subgroup), Subgroup 2 (solv.-ethanol subgroup), Subgroup 3 (solv.-acetone subgroup), Subgroup 4 (solv.-mixture subgroup), Subgroup 5 (solv.-detergent subgroup), Subgroup 6 (solv.-gradient subgroup), Subgroup 7 (ultr.-saline subgroup), Subgroup 8 (ultr.-ethanol subgroup), Subgroup 9 (ultr.-acetone subgroup), Subgroup 10 (ultr.-mixture-20 subgroup), Subgroup 11 (ultr.-mixture-40 subgroup)

There was a significant difference in ΔP among other subgroups with solv.-saline subgroup (*P* = 0.00), except for ultr.-saline subgroup (*P* = 0.39). There was no significant difference in ΔP among solv.-acetone, mixture, detergent subgroups (*P* = 0.93). For solv.-ethanol and ultr.-ethanol subgroups, solv.-acetone and ultr.-acetone subgroups, solv.-mixture and ultr.-mixture-20 subgroups, ΔP were all significant differences (*P* = 0.00). There was a significant difference in ΔP between ultr.-mixture-20 and ultr.-mixture-40 subgroups (*P* = 0.00) (Figs. 7, 8).

## Discussion

In this study, the efficacy of chemical solvent soaking treatments and ultrasonic cleaning methods on defatting of cancellous bone slices were investigated. Our results demonstrated that acetone was more efficient than 99% ethanol and gradient alcohol. Sharp et al. (1990) also claimed that alcohol was not a highly effective fat solvent. Contrarily, acetone has been proven to be a promising efficiency and is commonly used as a classic defatting solvent (Hua et al. 2020; Zhang et al. 2014). This is consistent with our experiment results. Additionally, Hua et al. (2020) investigated the defatting efficiency of acetone, gradient alcohol, and high-pressure washing on cancellous bone specimens obtained from human femoral condyle. Although the results demonstrated that there was no significant difference in the defatting efficiency for these three methods, the data still indicated that acetone was slightly better than alcohol in residual lipid content (acetone, 1.13% ± 0.22% vs ethanol, 1.28% ± 0.07%). In the study of Hua et al. (2020), a mechanical stirrer was used for mixing in the process of defatting with alcohol and acetone, whereas not in our study. This may be one of the reasons for the difference in our experiment results.

Typically, chloroform/methanol (Burke et al. 2017; Kalus et al. 2005) and trichloroethylene solution (Ta et al. 2005; Wear et al. 2017) are also frequently used for defatting in the literature. But, the toxicity of chloroform and trichloro-ethylene solution cannot be ignored. Animal experiments have been confirmed that exposure to chloroform would damage the liver and kidneys, and its toxicity would increase with time and dose (Morcos et al. 2015). Trichloro-ethylene induces cancer in rats and mice, and its metabolites are known to be toxic for the liver, kidneys, and lungs (Bruckner et al. 1989). As the chemical organic solvent, acute and durable exposure to ethanol and acetone would irritate eyes, nose, throat, as well as related to neurological symptoms (Arts et al. 2002; Le Dare et al. 2019). Nevertheless, compared to chloroform and trichloroethylene, they are considered of relatively low toxicity. Low levels of acetone are normally present in the body, and it is metabolized to harmless chemicals in the liver (Hansen et al. 1994). Similarly, ethanol is also widely used both in homes and in industries (Pendlington et al. 2001). In this experiment, our results indicated that the mixture of 99% ethanol and acetone (24 h, v/v = 1:1), and the combination of 99% ethanol (20 h) and detergent solution (4 h, v/v = 1:20) all could achieve ideal defatting efficacy for 2 mm thick cancellous bone specimens. Appearance observation demonstrated that the defatted bone specimens appear white, and the porous structure was clean.

Mechanical methods were primarily based on high-pressure water/gas jet (Sharp et al.^1990^; Zioupos et al., 2008) and ultrasonic cleaning (Shao et al. 2010; Sharp et al. 1990). The application of high-pressure water/gas on small specimens has certain limitations, easily lead to loss of specimens and contamination of the laboratory environment. Conversely, ultrasonic cleaning is an alternative method. However, our results demonstrated that the combination of ultrasonic cleaning and 0.9% saline solution could not achieve the goal of defatting. There was no significant difference between solv.-saline subgroup (0.9% saline solution, 24 h) and ultr.-saline subgroup (0.9% saline solution, ultrasonic bath, 20 min). In the short time, ultrasonic cleaning did not show any significant advantages when 0.9% saline solution was used as the medium. The results can be explained that the lipids, as nonpolar molecules, cannot be dissolved in polar 0.9% saline solution. Organic solvents, including 99% ethanol and acetone, can extract the lipid components from holes of cancellous bone. But the combination of ultrasonic cleaning and organic solvents (99% ethanol and acetone, 20 min or 40 min) still cannot achieve the same efficiency as chemical solvents soaking for 24 h. A possible explanation is that the dry lipid forms a barrier on the surface of the specimen, preventing the penetration and dissolution of organic solvents. Dried lipids cannot be removed in a short time with ultrasonic cleaning, whereas long exposure time in the chemical soaking group may not have this problem. The poor wettability of the specimen is an important factor affecting the efficiency of defatting (Frayssinet et al. 1998; Kalus et al. 2005). According to our results, there was a significant difference between ultrasonic cleaning for 20 min and 40 min using the same chemical solvent (99% ethanol mix acetone, v/v = 1:1) (*P* = 0.00). It seems to indicate the prolonged time is of great significance for improving the efficiency of ultrasonic cleaning in defatting of cancellous bone specimens. Sharp et al. (1990) used different reagents combined with an ultrasound bath to defat for cancellous bone specimens obtained from cadaveric femoral heads. The results indicated that alcohol in an ultrasound bath for 4 h does not remove all of the fat, trichloroethylene for 1 h partially removes fat, for 4 h removes all the fat in all the specimens. Nevertheless, they did not discuss the change in water temperature during the ultrasound bath.

The extension of ultrasonic cleaning time is beneficial to remove the lipids filled in the cancellous specimens, but the increase in water temperature with ultrasonic activity is indeed a factor that must be considered. Water temperature that is too high will directly cause cell damage and influence the mechanical properties of cancellous bone specimens (Yan et al. 2007; Yarmolenko et al. 2011). As water temperature increases with ultrasound activity, it is difficult to extend the ultrasonic cleaning time to 4 h in order to further verify the defatting efficiency of the combination of chemical reagents (99% ethanol and acetone) and ultrasonic bath. However, the rise of water temperature during ultrasonic cleaning was recorded in detail (Fig. 3). This is an important reference that can provide valuable information for future research related to ultrasonic cleaning.

Our study also has a few limitations. First, all specimens are from the same anatomical site (human femoral condyle). The porosity of cancellous bone specimens may vary if obtained from different locations of the body. For this study, cylindrical cancellous bone specimens obtained from multiple locations of the femoral condyle and a series of 2 mm thick bone slices were tested in order to minimize deviation in our study. This allows better comparability between the different defatting techniques but also decreases validity to other bones. It is likely, though, that the findings of the present study will also apply to cancellous bone samples from other metaphyseal regions. Second, only small specimens were investigated in our experiments. The defatting efficiency of the tested defatting methods on larger cancellous bone specimens needs to be further verified in the future. Third, slight changes in the density of the liquid medium related to the room temperature or the time used are all possible causes of deviation, especially for small and light specimens. Even in light of the mentioned limitations to our experiment, we believe that the results of this study can offer a valuable reference for the selection of defatting methods.

## Conclusion

The combination of 99% ethanol and detergent solution (v/v = 1:20) and the mixture of 99% ethanol and acetone (v/v = 1:1) seem to be the optimal defatting methods for 2 mm thick cancellous bone slices due to their effectiveness, availability, low-cost and safety. The experimental results provide effective, convenient, safe, and economical defatting approaches for small cancellous bone specimens. Chemical soaking for 24 h is more effective than ultrasonic cleaning with 99% ethanol or acetone for 20 or 40 min.

## Data Availability

The data used and analyzed during the current study are available from the corresponding author on reasonable request.
